# There Is Treasure Everywhere: Reductive Plastid Evolution in Apicomplexa in Light of Their Close Relatives

**DOI:** 10.3390/biom9080378

**Published:** 2019-08-19

**Authors:** Eric D. Salomaki, Martin Kolisko

**Affiliations:** Institute of Parasitology, Biology Centre Czech Acad. Sci., České Budějovice 370 05, Czech Republic

**Keywords:** apicoplast, Chromerid, Apicomplexa, Corallicolids, plastid reduction

## Abstract

The phylum Apicomplexa (Alveolates) comprises a group of host-associated protists, predominately intracellular parasites, including devastating parasites like *Plasmodium falciparum*, the causative agent of malaria. One of the more fascinating characteristics of Apicomplexa is their highly reduced (and occasionally lost) remnant plastid, termed the apicoplast. Four core metabolic pathways are retained in the apicoplast: heme synthesis, iron–sulfur cluster synthesis, isoprenoid synthesis, and fatty acid synthesis. It has been suggested that one or more of these pathways are essential for plastid and plastid genome retention. The past decade has witnessed the discovery of several apicomplexan relatives, and next-generation sequencing efforts are revealing that they retain variable plastid metabolic capacities. These data are providing clues about the core genes and pathways of reduced plastids, while at the same time further confounding our view on the evolutionary history of the apicoplast. Here, we examine the evolutionary history of the apicoplast, explore plastid metabolism in Apicomplexa and their close relatives, and propose that the differences among reduced plastids result from a game of endosymbiotic roulette. Continued exploration of the Apicomplexa and their relatives is sure to provide new insights into the evolution of the apicoplast and apicomplexans as a whole.

## 1. Introduction

Apicomplexa is a group of obligatory host-associated protists, with the majority of their diversity being represented by intracellular parasites. The most well-known Apicomplexa include the causative agents of devastating human and animal diseases, for example, malaria (*Plasmodium faliciparum*) and toxoplasmosis (*Toxoplasma gondii*). This diverse group has been projected to contain more than 1 million species; however, less than 1% have been described to date [1]. Apicomplexa is further divided into five groups: Piroplasmida (e.g., *Babesia*), Coccidia (e.g., *Toxoplasma*), Haemosporidia (e.g., *Plasmodium*), Cryptosporidia (e.g., *Cryptosporidium*), and Gregarina. Most Apicomplexa have many unique genomic modifications that facilitate their biology and propensity to an intracellular lifestyle; however, Gregarines provide a fascinating contrast as they primarily inhabit the gut of diverse invertebrates from terrestrial, freshwater, and marine habitats [2].

One of the most intriguing apicomplexan features is their reduced plastid organelle called the apicoplast (**Apico**mplexan **plast**id). All plastids originated from a primary endosymbiotic event in which a cyanobacteria was incorporated into a eukaryotic host cell, giving rise to the Archaeplastida (Glaucophyta, Chlorophyta, and Rhodophyta) [3]. Secondary and tertiary endosymbiotic events spread plastids throughout the eukaryotic tree of life as eukaryotes incorporated both “red” and “green” derived plastids from other photosynthetic eukaryotes [3]. Primary endosymbiosis resulted in the formation of two membranes around the plastid that originated from the cyanobacterial inner and outer membranes, while secondary and tertiary events resulted in two, three, or four membranes surrounding the plastids [4,5,6,7]. To date, all investigated Apicomplexa retain four membranes [8,9,10,11], with the inner two corresponding to the original cyanobacterial membranes, while the third and fourth are believed to originate from the endosymbiont and host, respectively, but work remains to conclusively demonstrate their origins [4,12].

During the transition from endosymbiont to organelle, many genes in the endosymbiont genome are transferred to the host nucleus, a process called endosymbiotic gene transfer. Once incorporated, the products of these genes are expressed in the nucleus, translated in the cytosol, and imported into the plastid organelle, resulting in host control of, and reliance upon, plastid functions. The intimate association between the apicoplast and the Apicomplexa has also been observed morphologically, as the shape of the apicoplast and its location in the cell change throughout the life cycle [13,14]. Based on its complicated and continually debated origin [15,16], and because it is essential for the survival of the Apicomplexa [17], this organelle has fascinated researchers since its discovery [18], from both an evolutionary perspective and as an important target for drug development against apicomplexan-caused diseases. Over the past ten years, several new lineages of Apicomplexa and their close relatives have been discovered [15,19,20,21]. Genomic and transcriptomic analyses of these taxa have shed light on the evolutionary history and function of the apicoplast, as well as broadened (and perhaps convoluted) the picture of apicomplexan evolution as a whole.

## 2. Historical Perspective

The apicoplast is an inconspicuous organelle that, although first observed in the 1960’s and 1970’s [22], took a long time to be recognized as a remnant plastid [18]. The initial evidence for the presence of a plastid in Apicomplexa was the discovery of two small chromosomes in *Plasmodium*: one ~6 kb and a second ~35 kb [23,24]. Gene mapping and sequencing of the chromosomes revealed that the 6 kb chromosome corresponds to the mitochondrial genome [25], whereas the 35 kb molecule is undoubtedly a remnant plastid genome based on its gene content, architecture [24], and localization to a four membrane-bound organelle [18]. Early phylogenetic analyses of plastid-encoded and nuclear-encoded genes have provided conflicting evidence for whether the plastid is of a “green” or “red” origin [26,27,28,29].

The functions of the apicoplast were not resolved by sequencing plastid genomes, as it only retained genes functioning in genome maintenance (replication and transcription) and two additional genes, *suf*B and *clp*C (see below) [30,31,32]. Instead, the initial predictions of apicoplast metabolism resulted from investigations of apicomplexan nuclear genomes and identification of the nuclear-encoded components of four putative plastid pathways: heme, isoprenoid, iron–sulfur cluster, and fatty acids biosynthesis [33,34]. These predictions were later confirmed by experimental evidence [35].

Today, the apicoplast has been found in all Apicomplexa lineages, with the exception of *Cryptosporidium*, which is one of only two lineages known to have completely lost its plastid [36]. There are conflicting reports on whether or not the gregarines possess a plastid; however, they remain relatively understudied compared to other apicomplexan lineages. There have been multiple publications indicating that no plastid exists in gregarines [37]. Interestingly, there is one conspicuous image showing what is described as a ‘vacuole’ containing three membranes in *Selenidium pendula* [38], which may be evidence of a gregarine apicoplast. Unfortunately, there is limited sequence data available for gregarines and no other evidence of a functioning plastid.

## 3. Apicomplexan Relatives and the Origins and Evolution of the Apicoplast

**Figure 1:**
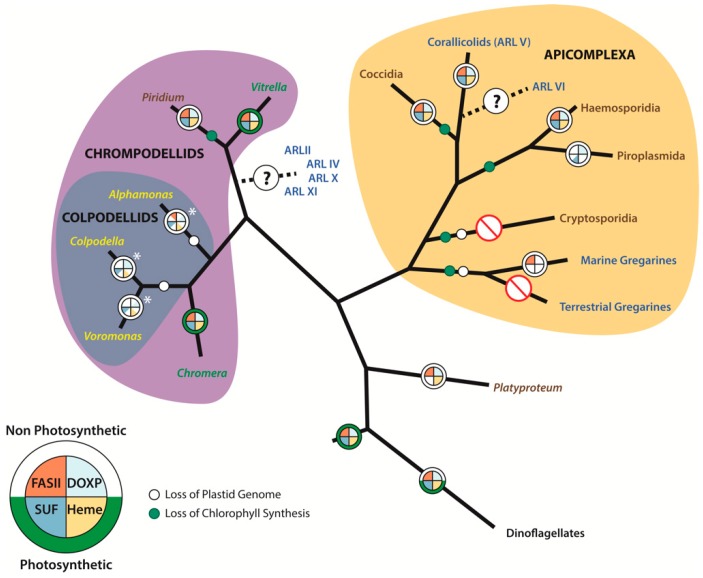
Phylogeny of the Apicomplexa and their close relatives representing the evolutionary history of their plastids and transitions in their nutrient acquisition strategies. Taxa labeled in brown are parasitic, yellow are free-living heterotrophs, green are autotrophic, and the nutritional strategies of taxa labeled in blue remain unclear. Plastid metabolic capacity is represented as a pie chart, with the Type II fatty acid synthesis pathway being represented in orange, the MEP/DOXP pathway represented in light blue, heme synthesis in gold, and the SUF pathway in dark blue; sections left white are missing from the plastid metabolism in the lineage. The ring around the metabolism pie chart is green if the lineage is photosynthetic, or white if non-photosynthetic. White circles on branches indicate the loss of a plastid genome, and green circles indicate the loss of chlorophyll biosynthesis. The asterisk next to Colpodellid plastid diagrams represents the current uncertainty regarding the presence of a plastid in these taxa.

Over the past decade, there have been several newly discovered lineages related to the Apicomplexa that have impacted our understanding of the evolutionary history of the apicoplast (Figure 1). Furthermore, the drastic decrease in the cost-per-base for sequencing with the advent of next generation tools has enabled non-model organisms to participate in the ‘-omics era’, yielding a significant amount of data from apicomplexan relatives that has increased our understanding of the evolution and function of the apicoplast. The following sections will discuss several of these lineages and how they have advanced our understanding of the apicoplast.

### 3.1. Chromerids

The first major finding regarding the evolutionary origin of the apicoplast occurred in the late 2000’s with the exciting discovery of two new lineages of single-celled algae: *Chromera* and *Vitrella*. Phylogenetic analyses placed these taxa as the closest known lineages to Apicomplexa, and surprisingly, both maintained fully functional photosynthetic plastids [15,19]. *Chromera* was initially isolated from a stony coral, *Plesiastrea versipora*, in Australia [19], and has been shown to be capable of infecting the larvae of other corals [39]. The nature of the relationship between *Chromera* and its hosts has been difficult to tease apart. Once considered a mutualist or facultative opportunist [40], a recent transcriptomic survey of *Chromera*-infected corals questioned the validity of that relationship [41]. Briefly, larvae of the coral *Acropora digitifera* were infected with *Chromera* and the coral transcriptomes were sequenced at three timepoints post-infection [41]. The coral was shown to respond quite differently when infected with *Chromera* than it does when infected by the mutualist, *Symbiodinium* [42], with the *Chromera-*infected *A. digitifera* transcriptome showing signs of a suppressed immune response, along with modified apoptotic machinery and phagosome maturation [41]. These results led researchers to conclude that the relationship between *Chromera* and its hosts is definitely not mutualistic; rather, it is likely either commensalic or parasitic [41].

*Vitrella brassicaformis* was isolated from a stony coral, *Leptastrea purpurea*, at the Great Barrier Reef by Andersen and Moore, and was initially studied under the isolate number CCMP1355 prior to being formally described [43]. It is important to note that while *Vitrella* and *Chromera* are commonly mentioned together in the literature dealing with apicomplexan evolution, they are very different organisms with different plastid and nuclear genomes, as well as very different plastid pigment profiles [15,43,44].

The discovery of Chromerids had an unprecedented impact on our understanding of apicomplexan evolution [44]. By placing *Chromera* and *Vitrella* as sister to the apicomplexans, the evolutionary origin of the apicoplast started to seem clear. Eventually, the *Chromera* and *Vitrella* plastid genome sequences allowed for phylogenetic analyses that clearly demonstrated that they are of a red algal plastid origin [15]. Transmission electron microscopy demonstrated that, like apicoplasts, these plastids are surrounded by four membranes [5]. Furthermore, these data verified that the plastids of dinoflagellates and Apicomplexa share a common origin, which, due to the lack of overlapping genes between the two groups of plastids, was previously impossible [15].

Recently, Fussy and Obornik [5] proposed the possibility that Chrompodellids and Apicomplexa underwent a tertiary endosymbiotic event, acquiring a plastid from an Ochrophyte alga, which replaced the original plastid that shared a common origin with dinoflagellates. This hypothesis is based on a recent deep phylogenomic analysis of plastid-encoded proteins that shows a propensity for *Vitrella* to branch within Ochrophyta [45]; yet, both Chromerid algae and Ochrophytes constitute rather long branches and the phylogenies might therefore be plagued by long branch attraction artifacts. There is, however, other evidence: (1) There are curious similarities in the plastid pigments of Chromerids and Eustigmatophytes (a subgroup of ochrophytes), for example, *Vitrella* has an almost identical pigment profile as the Eustigmatophyte alga *Nannochloropisis*; and (2) there are differences between apicomplexan and dinoflagellate plastids, most notably the number of plastid membranes and the plastid protein import machinery (see Fussy and Obornik for a detailed review) [5]. To reconcile this issue, a more detailed understanding of other plastid-bearing lineages within the Chrompodellids-Apicomplexa clade is essential, especially those from some other recently discovered lineages [21,46].

### 3.2. Colpodellids

Colpodellids are predatory heterotrophic flagellates that utilize myzocytosis—a process in which the predator punctures a small hole in the prey cell membrane and sucks out the cellular content [47,48]. Based on a phylogeny of ribosomal small subunit (SSU) genes, Colpodellids form a closely related lineage to the Apicomplexa and Chromerids [19,49], but do not possess a visually detectable plastid. This provides a fascinating evolutionary framework where three closely related organisms all had extremely different lifestyles and possessed surprisingly different plastid states—intracellular parasites with remnant plastids, free-living photosynthetic algae, and free-living non-photosynthetic specialized predators of other microbial eukaryotes.

Transcriptomic data from three Colpodellid genera—*Voromonas*, *Alphamonas*, and *Colpodella*—have recently become available [50,51]. A phylogenomic analysis of nuclear-encoded genes placed them into a strongly supported clade with *Vitrella* and *Chromera*, creating the group that is informally referred to as Chrompodellids [51]. To date, there remains no evidence of plastid genomes or microscopic evidence of the organelle within Colpodellids [51]. However, transcriptomic analyses have shown that Colpodellids possess nuclear-encoded genes for classical apicoplast pathways—isoprenoid synthesis, heme synthesis, type II fatty acid synthesis (FASII), and iron–sulfur (Fe–S) cluster synthesis—and surprisingly, a bipartite plastid-targeting signal has been detected on a number of proteins, lending some support to the presence of a remnant plastid organelle lacking a genome [51].

### 3.3. Corallicolids

The Corallicolids group was, until recently, a “mystery” lineage that was known exclusively from environmental sequences which could not be linked to a known organism. Janouskovec et al. [51] surveyed environmental 16S sequences in an attempt to identify misannotated plastid 16S genes. Indeed, they found nearly 10,000 sequences predominately called ‘novel bacteria’ that instead belonged to a diverse array of plastid-bearing lineages across the tree of life. Most interesting was the discovery of several new lineages closely related to Apicomplexa that they denoted as ARL-I through ARL-VIII [46]. Initially, it was possible to assign some of these lineages, as *Chromera* falls within ARL-III and *Vitrella* was associated with ARL-I. However, additional lineages were completely novel, representing undescribed organisms. ARL-V was a lineage of particular interest as it was shown to be closely related to Apicomplexa and was associated with a minimum of 16 coral species. At the time, it was proposed to be an apicomplexan parasite of corals, but further research has shown that ARL-V sequences are mostly recovered from phototrophic zones, suggesting that the lineage is photosynthetic [52]. Recently, Kwong et al. [20] conclusively linked the ARL-V lineage to a group called Genotype-N, an apicomplexan known from 18S environmental sequencing [53].

### 3.4. New Parasitic Lineages–Piridium and Platyproteum

*Piridium* and *Platyproteum* are two poorly studied genera of intracellular parasites that were previously classified as Apicomplexa—*Piridium* as a Schizogregarine [54] and *Platyproteum* as an Archigregarine [55]. Cavalier-Smith [56] proposed, based on SSU rRNA genes and morphology, that *Platyproteum* is related to Perkinsida. Interestingly, a recent phylogenomic analysis [57] resolved these species as two independent lineages, separate from Apicomplexa. *Piridium* forms a branch sister to *Vitrella*, and *Platyproteum* is a sister lineage to the whole clade of Apcomplexa-Chrompodellida.

## 4. Plastid Genome Evolution within the *Platyproteum*-Chrompodellids-Apicomplexa Lineage

**Figure 2:**
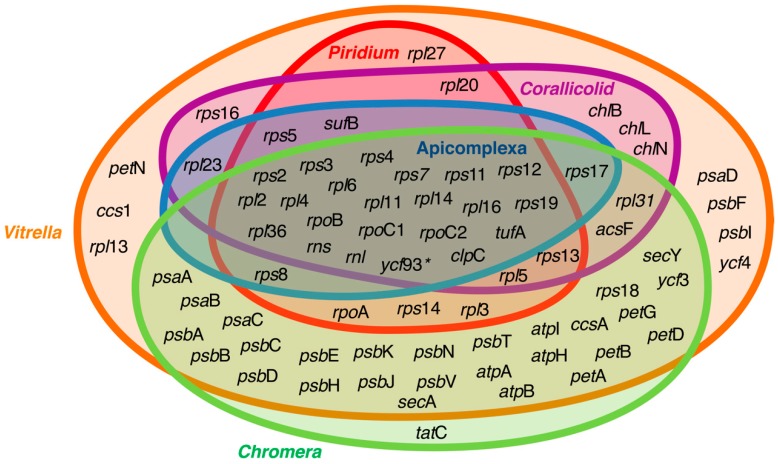
Venn diagram representing genes retained in the plastid genomes of apicomplexans and their close relatives. The overlapping rings show the core genes retained by the apicoplast, which primarily encodes genes for translation and replication, as well as *suf*B and *clp*C, which have been suggested to be essential for genes preventing plastid genome loss. The asterix next to *ycf*93 signifies that the presence of this fast-evolving gene has not been explored in all lineages depicted in the diagram. Data for the figure were collected from Janouskovec et al., Mathur et al. [15,57], and the National Center for Biotechnology Information (NCBI) GenBank database.

The apicoplast genome is one of the most reduced plastid genomes known, both in size and gene content (Figure 2), with the exception of some dinoflagellate plastids that are even more reduced in gene content [58,59]. This indicates that most of the genes from the endosymbiont were either transferred to the nucleus or lost. The typical apicoplast genome is ~35 kb long and contains approximately 50 genes, including rRNAs and tRNAs [31,32,60], with the vast majority of these genes functioning in genome maintenance (i.e., replication, transcription, and translation). The only genes that function outside genome maintenance are *suf*B and *clp*C. The *suf*B gene is a critical part of the sulfur activation pathway for iron–sulfur cluster assembly (see below) and is therefore essential for a properly functioning SUF pathway, while the function and importance of *clp*C remains unknown; however, it is presumed to be involved in protein import or to work as a substitute for the chaperone *hsp70* [61].

Interesting patterns appear when apicoplast genomes are compared to the plastid genomes of their close relatives. Both photosynthetic relatives of apicomplexans, *Chromera* and *Vitrella*, despite being photosynthetic, possess highly reduced plastid genomes (having 57 and 73 protein coding genes, respectively; Figure 2) compared to red algae and other red-algal derived plastids [15]. For example, there are approximately 200–250 genes in a standard photosynthetic red algal plastid [62], and even the recently described reduced plastids of non-photosynthetic parasitic red algae retain 70–85 genes [63,64]. Interestingly, *Piridium*, which is a parasitic lineage sister to *Vitrella* to the exclusion of apicomplexans, contains a plastid genome that is almost indistinguishable from the plastid genome of Apicomplexa, suggesting very strong evolutionary constraints and convergence in the genome reduction process [57].

The Corallicolids (previously ARL-V) are one of the more fascinating recent discoveries, as they retain chlorophyll biosynthesis genes in their plastid genome, representing an intermediate state between *Vitrella* and a typical apicoplast [20]. Moreover, nuclear-encoded rRNA-based phylogenies place Corallicolids as a sister lineage to coccidia (see above), which implies multiple independent losses of chlorophyll biosynthesis within Apicomplexa [20].

Apicoplast genomes also contain inverted repeats that mainly consist of an rRNA operon that appears to be ancestral since they are characteristic of many plastid genomes [30,32,60,65]. These inverted repeats have likely been lost twice independently in the evolution of this lineage, once in *Chromera*, which has also lost the typical circular structure and exists as a linear molecule that possesses inverted repeats of three genes at the end (*orf*264, *psb*A, and *atp*H2) [15], and in *Piridium*, where a potential remnant of the inverted repeat can be detected as a putative ribosomal large subunit (LSU) pseudogene [57].

Most plastids utilize a canonical genetic code, but the plastid genomes of Coccidia, *Piridium, Chromera*, and *Corallicolids* use an alternative genetic code, where the codon UGA encodes Tryptophan instead of the usual translation termination codon [15,20,57,61]. The distribution of this alternative code usage throughout these taxa suggests that there were at least three independent origins of the non-canonical genetic code within the *Platyproteum*-Chrompodellids-Apicomplexa clade. This frequency suggests a tendency among rapidly evolving plastids to switch to a non-canonical genetic code.

## 5. Apicoplast Metabolism

The typical apicoplast partakes in four metabolic pathways: heme biosynthesis, the FASII pathway, the SUF pathway for Fe-S cluster synthesis, and the 2-*C*-methyl-d-erythritol 4-phosphate/1-deoxy-d-xylulose 5-phosphate (MEP /DOXP) for isoprenoid synthesis. Most of the proteins functioning in the apicoplast are nuclear-encoded and have to be imported post-translationally. Therefore, the apicoplast retains the typical import pathways [66]—the plastidial TIC (translocon of the inner membrane) [67] and TOC (translocon of the outer membrane) [68] import machinery, as well as so-called SELMA [69,70,71], which is modified endoplasmatic reticulum-associated degradation machinery. Proteins targeted to the apicoplast possess the bipartite targeting signal consisting of the signal peptide and the targeting peptide.

### 5.1. Heme Synthesis Pathway

Heme is a prosthetic group of many proteins that allows for the binding and carrying of small molecules or electrons and is an essential part of fundamental structures, including chlorophylls and hemoglobin. Heme biosynthesis is a very convoluted pathway in Apicomplexa, as it takes place in three different cellular compartments: the mitochondrion, the cytosol, and the apicoplast. Heme synthesis is performed by a series of eight enzymatic reactions: the first reaction occurs in the mitochondrion, followed by a sequence of four reactions localized in the apicoplast, then one reaction in the cytosol, and ending with the last two steps back in the mitochondrion [66,72,73,74]. In most photosynthetic eukaryotes, the heme pathway is completely localized to the plastid [75]; however, in *Chromera*, the first step has been transferred to the mitochondrion. It is common for several steps in the plastid heme synthesis pathway to be performed by several different enzymes with different endosymbiotic origins (i.e., versions of the enzyme of cyanobacterial origin, versions from the primary algal nucleus, and versions from the secondary host nucleus) [75]. In Apicomplexa, only one homolog per enzymatic reaction has been retained in the apicoplast and the last three or four steps have been transferred to the cytosol and mitochondrion [66,75].

Heme biosynthesis is essential for certain developmental stages of *Plasmodium*, specifically for the liver and *Anopheles* stages of the life cycle. Heme is necessary for maintaining a properly functioning electron transport chain in the mitochondria. In *Plasmodium*, this pathway is active during the blood stages, but it is not essential, since heme is readily available from the surrounding environment [76]. In actuality, *Plasmodium* parasitizes red blood cells, so there is a large amount of heme from the host that can be processed by the parasite and stored in the form of hemozoin [77]. Indeed, experiments have shown that both sources of heme, from the host and its own synthesis, are recruited to the *Plasmodium* mitochondria and converted to hemozoin [76].

### 5.2. Type II Fatty Acid Synthesis Pathway

The type II fatty acid synthesis (FASII) pathway is a widespread pathway in prokaryotes and plastids of plants and algae, and was first characterized in *Plasmodium falciparum* and *Toxoplasma gondii* in 1998 [33]. This study identified the nuclear-encoded proteins *acp*P, *fab*H, and *fab*Z, and demonstrated that the products of these genes are targeted to the apicoplast [33]. The role of fatty acids in a variety of essential cellular processes and the finding that the FASII pathway could be targeted for therapies without impacting the eukaryotic Type I fatty acid synthesis (FASI) pathway led to a wealth of research on these genes [33,78,79]. Only later was it recognized that the *Theileria parva* genome did not encode any genes for the FASII pathway [80] (Figure 1), and that the FASII pathway was only essential for the liver stage and sporozoite development in the *Anopheles* part of the life cycle of *Plasmodium* [81,82], suggesting that drugs targeting the FASII pathway would mostly serve for prophylaxis, rather than as cures for the actual diseases.

FASII has also been found to be missing in *Cryptosporidium parvum*; however, an alternative pathway (FASI) has been detected in the *C. parvum* genome that is capable of fatty acid elongation [83,84,85]. Piroplasmida, including *T. parva* and *Babesia bovis,* as well as *Platyproteum,* appear to have lost the fatty acid synthesis pathway entirely [57,80,86]. Intriguingly, a complete FASII pathway was recovered from the transcriptomes of two marine gregarines, *Lecudina* and *Pterospora*, but it appears to be lost from the sister clade of terrestrial gregarines [57]. This wider taxonomic sampling has demonstrated that, although the FASII pathway is retained in Coccidia (e.g. *Toxoplasma*) and Haemosporidia (e.g., *Plasmodium*), it is not essential for the maintenance of the apicoplast and has been independently lost a minimum of four times in the *Playproteum*/Chrompodellids/Apicomplexa clade.

### 5.3. MEP/DOXP Pathway

Isoprenoids are important lipid compounds that are used, for example, in tRNA modifications and the prenylation of proteins, and function as a precursor for dolichols and ubiquinone. There are two pathways for the synthesis of the precursors of isoprenoids: the eukaryotic mevalonate pathway and the prokaryotic MEP/DOXP pathway. The MEP/DOXP pathway consists of seven steps, the inputs are pyruvate and glyceraldehyde-3-phosphate, and it functions to synthesize dimethylallyl pyrophosphate (DMAPP) or isopentenyl pyrophosphate (IPP)—the building blocks of isoprenoids [77].

Dependency on the isoprenoid pathway is still not fully resolved; however, it has been shown that antibiotic disruption of the apicoplast MEP/DOXP pathway leads to the death of blood stage *Plasmodium* [87], unless rescued by supplementing external IPP [88]. Moreover, *Plasmodium* that is maintained under antibiotic treatment to disrupt the apicoplast MEP/DOXP pathway, while simultaneously supplemented with IPP, appears to no longer localize nuclear-encoded proteins and over a few generations, completely loses the apicoplast organelle [88]. This demonstrates that, at least for the *Plasmodium* blood-stages, IPP is the only essential product of the apicoplast, and other apicoplast functions are there merely to support the MEP/DOXP pathway and apicoplast replication. While the actual role of the apicoplast-produced IPPs is not yet fully understood, they may function in providing IPPs for ubiquinones in the mitochondria [87], or be used for the prenylation of proteins [89,90].

### 5.4. SUF Pathway

Iron–sulfur clusters are common prosthetic groups that are essential for proper protein function in all lineages of the tree of life. The primary function of Fe–S clusters is to mediate electron binding and transport and they are therefore essential for redox reactions [91]. There are four known Fe–S cluster synthesis pathways: (1) the eukaryotic cytosolic iron-sulfur cluster assembly (CIA) pathway; (2) the prokaryotic nitrogen fixation (Nif) pathway; (3) the prokaryotic and typical mitochondrial “iron–sulfur cluster” (ISC) pathway; and (4) the prokaryotic and plastidial sulfur mobilization (SUF) pathway. The SUF pathway is the only one for which a gene is generally encoded by the apicoplast genome (*suf*B) [30,31,32]. This pathway is comprised of multiple components: *suf*S and *suf*E act as cysteine desulfurases, while *suf*BCD and *suf*A are assembly scaffolds and transporters [77,92]. Almost all proteins functioning in apicoplast metabolic pathways are encoded from the nuclear genome and subsequently imported into the apicoplast [34]. Therefore, iron–sulfur proteins are imported to the apicoplast as apoproteins, and there acquire their Fe–S cluster, becoming functioning holoproteins.

The SUF pathway supplies the Fe–S clusters for enzymes that produce isopentenyl pyrophosphate (IPP) [92], which has been recognized as an essential product of the apicoplast and perhaps the protein/pathway that prevents the loss of the apicoplast. Therefore, retention of the apicoplast genome appears to also be dependent on the SUF pathway, as the *suf*B gene is encoded by most apicoplast genomes.

## 6. Hidden Treasure

Our understanding of the variability in apicoplast metabolic capacity has improved immensely over the past decade. The advent of next generation sequencing, combined with the ability to obtain genomes [93,94] and transcriptomes [57,95,96] from single cells, has provided a glimpse into the evolution and metabolism of previously understudied and even unknown lineages [20,97,98]. The expanded exploration of Apicomplexa and apicomplexan relatives continues to provide a crucial understanding of the apicoplast, and Apicomplexa in general. Furthermore, every relative of Apicomplexa that has been studied has provided a new twist to an exciting story. This is underscored by the recent description of the Corallicolid plastid that retains chlorophyll biosynthesis genes, yet branches within the core members of the Apicomplexa [20], and the findings exploring plastid metabolism in the gregarines, *Piridium*, and *Platyproteum* [57].

The Corallicolids present an especially fascinating case. Initially recognized as plastid sequences of an apicomplexan relative from published 16S sequences misannotated as ‘bacteria’ [46], these data were recently linked to a novel apicomplexan lineage, likely sister to Coccidia (Figure 1). Like all other apicoplasts, the Corallicolid plastid genome lacks genes for the photosystem proteins, which strongly implies a non-photosynthetic plastid. However, genes involved in chlorophyll biosynthesis are retained and actively transcribed [20]. Based on the most recent phylogenetic analysis, the placement of the Corallicolids indicates that chlorophyll biosynthesis has been lost twice in the Apicomplexa [20] (Figure 1). The retention of chlorophyll biosynthesis genes in Corallicolids might make it attractive to suggest that photosynthesis was not lost at the common ancestor of Apicomplexa. Conversely, the fact that Corallicolids retain chlorophyll biosynthesis genes and remain non-photosynthetic makes the loss of photosynthesis in the ancestor of Apicomplexa the most parsimonious explanation. The role of the chlorophyll biosynthesis genes remains a mystery, but Corallicolids were also detected in two groups of non-photosynthetic corals, leading the authors to suggest that they may have developed a use for chlorophyll other than light harvesting [20].

*Piridium* was first described as a Schizogregarine nearly 90 years ago [54]; however, it remained largely unstudied. The first molecular phylogeny to include this taxon was only recently completed, placing it surprisingly and robustly outside of the core Apicomplexa clade and instead within the Chrompodellids as a sister lineage to *Vitrella* [57]. The placement of an assumed apicomplexan parasite sister to the photosynthetic *Vitrella* alone was surprising, but the plastid genome of *Piridium* was strikingly similar to an apicoplast. This indicates that *Piridium* and the core Apicomplexa convergently evolved a highly reduced plastid that retains the heme, FASII, MEP/DOXP, and SUF pathways, further supporting the hypothesis that a common path to plastid reduction exists within these lineages and that core pathways prevent total plastid loss.

## 7. What Gene Holds the Key to Apicoplast Retention?

Arguments persist regarding which genes or pathway(s) are the limiting factor(s) for total plastid genome and plastid organelle loss. The MEP/DOXP pathway has been implicated multiple times as the last holdout [51,88,99], as it appears throughout the plastid-retaining Apicomplexa and their relatives (excluding *Cryptosporidium* and the gregarines) (Figure 1). This scenario is reminiscent of the ISC pathway in highly reduced anaerobic mitochondria, where all other pathways can be lost, but the ISC pathway remains [100]. It is known that the ISC pathway provides an unknown metabolite necessary for the function of the cytosolic CIA machinery [101]. Interestingly, in the anaerobic protist *Monocercomonoides*, the ISC pathway has been replaced by the horizontally acquired SUF pathway and the remnant mitochondrion has been completely lost [102]. Similarly, if the MEP/DOXP pathway becomes obsolete (for example, a parasite manages to scavenge the necessary metabolites from the host, e.g., *Crytposporidium*), the plastid can be lost. However, this hypothesis has come into question considering the necessity of the SUF pathway for producing Fe–S clusters for enzymes involved in the synthesis of IPP [77,92]; therefore, plastid retention may hinge on both pathways rather than a single one.

Curiously, since the FASII and heme pathways appear to still be essential in certain life cycle stages of *Plasmodium* and *Toxoplasma* [81,82], selective pressure is maintained on both pathways. Oddly enough, neither the MEP/DOXP nor the SUF pathway was found in the transcriptome of marine gregarines; however, a complete FASII pathway was conserved. These findings are based on single-cell transcriptomes and it is plausible that the enzymes functioning in isoprenoid synthesis may not have been recovered due to an incomplete transcriptome, though this seems somewhat unlikely since the transcriptomes show relatively high coverage of the genome (based on BUSCO scores) and several lineages have been sequenced [57].

The widespread distribution of *sufB* and *clp*C genes suggests that retaining these genes in the plastid genome creates a plastid genome dependency in Apicomplexa and *Piridium*, thereby preventing genome loss. When these essential genes are transferred to the nuclear genome, as seen in Colpodellids, the plastid genome can be lost entirely [51]. The one notable exception is the apicoplast genome of piroplasmids, where *suf*B has been lost and as such, dependency on the plastid genome is likely exclusively due to the retention of *clp*C [30,60,103]. Both the mitochondrion and apicoplast need formylated initiator tRNA, encoded by the *trnf*M gene, which is missing in the mitochondrial genomes of apicomplexans. It has been proposed that an apicoplast-produced initiator tRNA is imported from apicoplasts to mitochondria [104], creating dependency on the apicoplast genome. However, this gene is also missing from the apicoplast genomes of Piroplasmida and most likely cannot be solely considered as essential. Another candidate gene for apicoplast genome dependency, *ycf*93, was recently discovered [51,105]. *ycf*93 was first characterized as a fast-evolving gene encoded by the *Plasmodium* apicoplast that retains a C-terminal transmembrane domain that is conserved in all apicomplexans; however, the presence of *ycf*39 in *Vitrella* is unclear [105]. Furthermore, the presence of *ycf*39 remains unconfirmed in the more recently explored lineages, including *Piridium, Platyproteum*, the Corallicolids, and marine gregarines.

These data indicate that there are multiple paths to plastid genome reduction. Perhaps plastid dependency is not simply a function of core pathways or genes, but instead, a product of the environment the organism is evolving in. This could even be seen as a game of endosymbiotic roulette when it comes to establishing which genes are retained in the plastid or the nucleus during the process of endosymbiotic gene transfer. There are three possible results for a gene during endosymbiosis: retention in the organelle, transfer to the host nucleus, and total loss. It is plausible that the genes that are retained in either the nucleus or the apicoplast in one lineage are different than those in another. For example, the *suf*B gene is maintained in the apicoplast genome of many Apicomplexa, creating positive selection pressure for plastid genome retention in these lineages. In others, this gene was transferred to the nucleus, which led to plastid genome loss, but the organelle itself was likely retained, with all its proteins being targeted from the nucleus (i.e., Colpodellids). Finally, it is possible that some organisms learned to scavenge all these metabolites from their environment, leading to complete loss of the pathway and eventually even the organelle (i.e., *Cryptosporidium*). Situations similar to this could all lead to different versions of a streamlined plastid genome and organellar functional capacity, where genes, pathways, and genomes are reduced or lost based on how easily the products of each can be acquired from the environment: the easier it is to acquire, the less likely it is that the particular gene, pathway, or genome will be retained. If a gene for an essential pathway is retained in the apicoplast, it will restrict complete loss of the plastid genome. Alternatively, genes for essential pathways that are transferred to the nucleus and targeted back to the plastid will shape the function of the plastid, but not necessarily restrict the plastid genome from being lost.

## 8. Conclusions

The wealth of data generated over the past few years has provided some exciting information and vastly improved our understanding of apicoplast evolution and metabolism. One of the most important takeaways from this is the importance of continued exploration and investigation of apicomplexan relatives. Basically, every relative of Apicomplexa that has been studied over the past decade has produced fascinating data and provided unique insights into apicoplast evolution and the biology of Apicomplexa, including potential targets for future drug therapies for some of the most widespread and harmful disease-causing parasites of humans and animals.

One of the major problems in understanding apicomplexan evolution is that data often do not overlap with other potential relatives; for example, the link between the apicoplast and that of peridinin-containing dinoflagellates had been hinted at, but was left unconfirmed, until *Chromera* and *Vitrella* were sequenced [15]. Additionally, these data established that the Apicomplexa evolved from a once photosynthetic ancestor and that the apicoplast is a highly-reduced, formerly photosynthetic plastid. However, while some questions have been answered, others have been raised. Are *Vitrella* and *Chromera* secondarily free-living? It is certainly within reason, given that the *Chromera* and *Vitrella* plastids retain an almost identical gene content, yet have a very different structure and produce different pigments. Furthermore, current phylogenies show that *Chromera* and *Vitrella* are nested within several parasitic species [57] (Figure 1). Potentially, these lineages arose as a result of independent acquisitions of secondary or tertiary plastids. These similarities and differences make it tempting to conclude that *Chromera* and *Vitrella* might be secondarily free-living, as much of the genetic material for maintaining a plastid could have remained present in the nuclear genomes, allowing straightforward independent plastid reacquisition. However, given that the nuclear genomes of *Chromera* and *Vitrella* are not reduced, as in the cases of apicomplexan parasites and *Piridium*, the only plausible scenario would be a reversal from a parasitic to free-living lifestyle very shortly after the transition to parasitism, before genome adaptation to the host began.

The journey continues with exciting findings in *Piridium*, demonstrating the convergent evolution of an ‘apicoplast’, as well as the Corallicolids and marine gregarines exhibiting massive variation from a traditional apicoplast. A major struggle when interpreting the available data and studies is that the most complete information is only available for the traditional apicomplexan parasites *Plasmodium* and *Toxoplasma*, while for all other lineages, it is very common that only transcriptomic or partial genomic data are available. Investigations of published environmental sequences and high-throughput amplicon data have uncovered numerous lineages of previously unrecognized relatives of the Apicomplexa that promise to be important additions to the evolutionary puzzle, but there is nothing known about them aside from the fact that they exist [21,46]. Are there other lineages waiting to be discovered and studied that will allow us to better understand the evolution of the apicoplast?

Continuing the investigation of these unexplored and novel lineages is essential. More data needs to be generated for the Corallicolids to confirm their position among Apicomplexa, pinpoint the role of the chlorophyll biosynthesis genes, and determine the exact function of their ‘apicoplast’ (is it really non-photosynthetic?). Increased morphological research, and the sequencing of full genomes and ‘apicoplast’ genomes, will provide a more concrete understanding of the apicoplasts in *Piridium, Platyproteum,* Corallicolids, and marine gregarines. Further investigations into the relatives of the Chrompodellids and Apicomplexa are essential and will surely shed light on some evolutionary mysteries while, at the same time, perplex us by raising new questions.
